# Endovascular thrombectomy for isolated posterior cerebral artery occlusion: distinct clinical presentation patterns and neurological outcomes in P1 versus P2 segments

**DOI:** 10.3389/fneur.2026.1807618

**Published:** 2026-04-21

**Authors:** Natalie van Landeghem, Daniel van Landeghem, Jordi Kühne Escolà, Benedikt Frank, Martin Köhrmann, Christoph Ziegenfuß, Johannes Harmes, Johannes Haubold, Isabel Wanke, Cornelius Deuschl, Michael Forsting, Yan Li

**Affiliations:** 1 Institute of Diagnostic and Interventional Radiology and Neuroradiology, University Hospital Essen, Essen, Germany; 2 Department of Neurology and Center for Translational Neuro- and Behavioral Sciences (C-TNBS), University Hospital Essen, Essen, Germany; 3 Department of Neuroradiology, Hirslanden Clinic, Zurich, Switzerland; 4 Institute of Diagnostic and Interventional Radiology, Neuroradiology and Nuclear Medicine, University Hospital Knappschaftskrankenhaus Bochum, Ruhr University Bochum, Bochum, Germany

**Keywords:** acute ischemic stroke, mechanical thrombectomy, neurological outcome, P1 segment, P2 segment, posterior cerebral artery

## Abstract

**Background:**

Endovascular thrombectomy (EVT) for isolated posterior cerebral artery (PCA) occlusion remains incompletely characterized, particularly regarding segment-specific presentation and outcomes. We aimed to compare clinical profiles, procedural results, and early neurological and imaging outcomes between P1 and P2 PCA occlusions treated with EVT.

**Methods:**

We retrospectively analyzed EVT-treated patients with acute ischemic stroke at a tertiary center from 2018–2025 (*n* = 1,166). Thirty-nine patients with isolated PCA occlusion were included in the final analysis (P1 *n* = 27; P2 *n* = 12). Successful reperfusion was defined as mTICI 2b–3. Baseline and 24-h CT were assessed for detecting ischemic change using posterior circulation ASPECTS (PC-ASPECTS). Neurological deficits were analyzed using NIHSS total scores and clinical domain profiles.

**Results:**

Patients with P1 occlusion presented with higher admission stroke severity than those with P2 occlusion [NIHSS 12 (7–23) vs. 7 (4–9)] and more frequent motor/sensory deficits and facial palsy, whereas P2 occlusions commonly presented with visual dysfunction. Successful reperfusion was achieved in 72% overall (P1 78% vs. P2 58%). Median 24-h PC-ASPECTS was similar [8 (IQR 8–9)]. Despite successful reperfusion in P1 occlusion, neurological impairment frequently persisted at discharge, particularly in motor, sensory, vigilance, and language-related domains. Infarct demarcation at 24 h remained frequent, including thalamic involvement in 41% (P1) and 36% (P2). For P1 occlusions, successful reperfusion was associated with less thalamic (29% vs. 83%) and occipital (52% vs. 83%) infarct demarcation. Any intracranial hemorrhage occurred in 13% without segment differences; in-hospital mortality was 26%.

**Conclusion:**

P1 and P2 segment occlusions showed distinct clinical phenotypes, while EVT was technically feasible with acceptable safety. However, persistent deficits and brain tissue injury were common despite successful reperfusion.

## Introduction

Posterior cerebral artery (PCA) infarctions account for approximately 5–8% of all ischemic strokes (1, 2). Despite their rarity, PCA strokes encompass a broad spectrum of anatomical involvement and clinical presentations. Depending on affected anatomical areas and collateral supply, patients may present with isolated visual field loss or with higher-order visual dysfunction like impaired reading and deficits in face or object recognition. Besides visual loss and possible memory dysfunction and neurobehavioral symptoms, proximal occlusion of PCA may also involve thalamic and mesencephalic regions, which can result in contralateral hemiparesis and/or hemisensory loss (3).

Due to this broad clinical presentation, accurate symptom recognition and severity assessment can be challenging in posterior circulation stroke. Compared to anterior circulation strokes, posterior events are more frequently missed or diagnosed with delay, particularly when symptoms are atypical (e.g., dizziness, nausea/vomiting, gait imbalance) or when deficits are predominantly visual–cognitive (4, 5). In addition, commonly used bedside scales such as the National Institutes of Health Stroke Scale (NIHSS) may underweight posterior circulation deficits. Posterior-circulation–adapted approaches such as the POST-NIHSS therefore have been proposed to improve diagnostic accuracy in this setting (6).

While endovascular thrombectomy (EVT) has evolved as a treatment option for anterior circulation large vessel occlusion, evidence for EVT in isolated PCA occlusion remains less mature and is largely observational. Studies like the TOPMOST and PLATO registries show that EVT for PCA occlusions is technically feasible and may provide early benefit. However, these studies have not uniformly demonstrated that EVT leads to better long-term functional outcomes than best medical management (7, 8). Importantly, the occlusion sites may matter: proximal P1 occlusions may show different clinical profiles including more frequent motoric and sensory impairment than P2 occlusions, which may predominantly manifest as disabling visual–cognitive syndromes that are not well reflected by global severity scales.

From the angiographic procedural perspective, thrombectomy of distal PCA occlusion can be technically demanding due to smaller vessel caliber, sharper vascular angulation, and the more distal access trajectory. Moreover, in routine clinical settings, the use of balloon guide catheters for protection against distal emboli and increased reperfusion success is often not feasible in posterior circulation because of small vessel caliber and permanent blood flow from contralateral vertebral artery. At the same time, the collateral physiology of isolated PCA occlusion is not well characterized. These considerations complicate patient selection, particularly when symptoms are severe but are mainly driven by deep perforator ischemia (e.g., thalamo-mesencephalic involvement), which may be less amenable to restoration by only recanalization of the parent vessel. Furthermore, systematic evidence on the extent to which patients with isolated PCA occlusion recover after reperfusion therapies—especially across different occlusion segments—remains limited.

Against this background, we aimed to investigate isolated PCA occlusions treated with EVT in real-world practice to compare clinical presentations, imaging, angiographic, and early clinical outcome profiles between P1 and P2 occlusions.

## Methods

### Study design and patient selection

Our study was approved by the local institutional review board (22-10821-BO) in accordance with the Declaration of Helsinki. Due to the retrospective nature of the study, the requirement for informed consent was waived. All data were extracted from a prospectively maintained stroke registry.

This retrospective single-center study included all consecutive patients with acute ischemic stroke treated with EVT at a tertiary comprehensive stroke center between January 2018 and October 2025.

**Figure 1:**
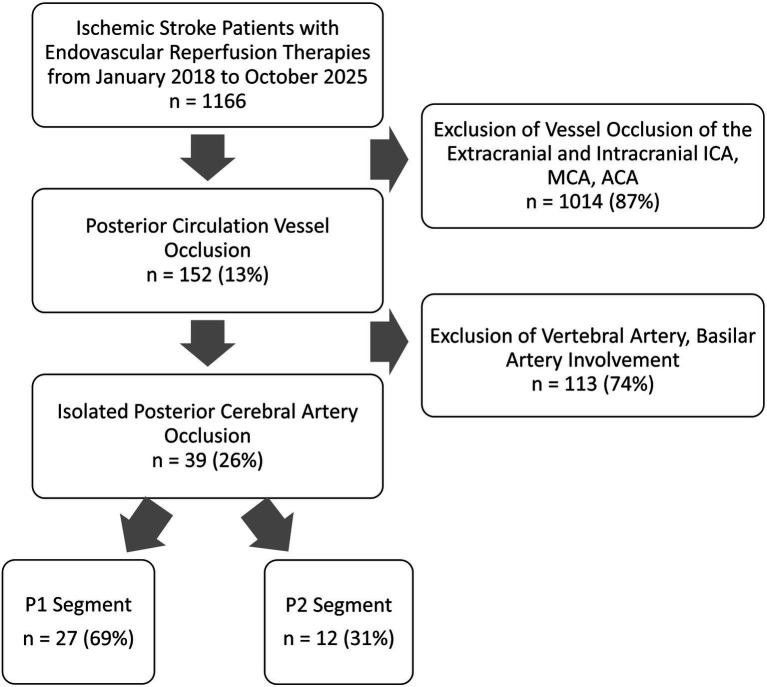
Flowchart of selection process. P1/P2, segments of the posterior cerebral artery; ICA, internal carotid artery; MCA, middle cerebral artery; ACA, anterior cerebral artery.

Patients were eligible for inclusion if they had an angiographically confirmed isolated PCA occlusion involving either the P1 segment or the P2 segments and underwent EVT. Patients with concomitant occlusions of other intracranial vessels or incomplete imaging and/or neurological data were excluded (Figure 1).

### Imaging assessment and occlusion classification

**Figure 2:**
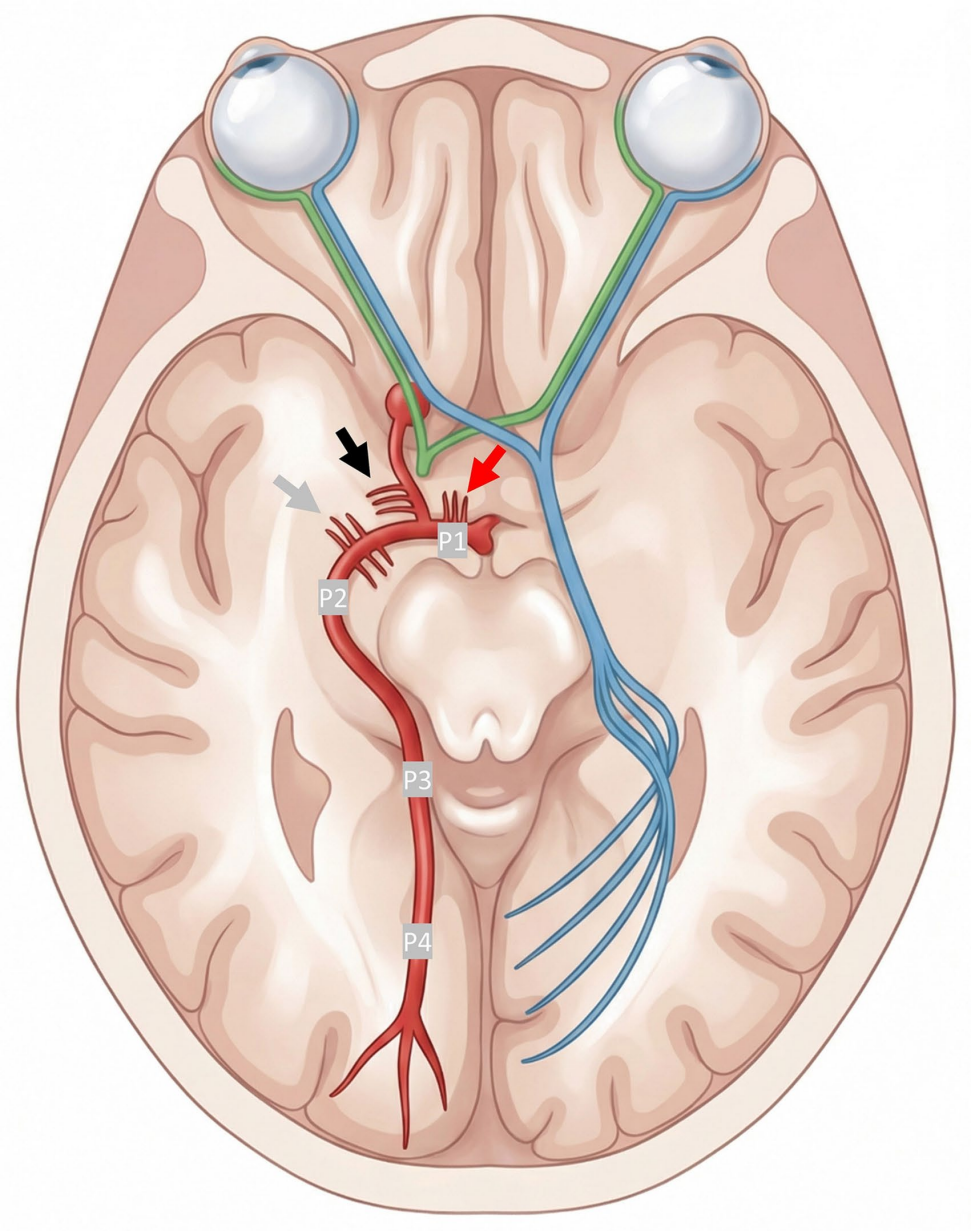
Posterior cerebral artery segments and optic radiation. Schematic illustration of the posterior cerebral artery with segmental classification into P1–P4 segments (right hemisphere) and the optic radiation (left hemisphere), highlighting the anatomical substrate of visual field deficits by ischemia in PCA territory. Perforating arteries arising from P1 segment (paramedian arteries, e.g., artery of Percheron, red arrow), from posterior communicating artery (polar arteries = tuberothalamic arteries, black arrow), and from P2 segment (inferolateral arteries = thalamogeniculate arteries, peduncular perforators, grey arrow) (33). PCA, posterior cerebral artery; P1–P4, segments of the PCA.

Baseline imaging consisted of non-contrast computed tomography (NCCT) and CT angiography, supplemented by CT perfusion (CTP) imaging when available. Occlusion site was classified on digital subtraction angiography (DSA) according to segmental PCA anatomy (Figure 2). The P1 segment was defined as the pre-communicating segment from the basilar apex to the junction with the posterior communicating artery, and the P2 segment as the post-communicating segment within the ambient cistern according to standard anatomical definitions (9).

Posterior circulation Alberta Stroke Program Early CT Score (PC-ASPECTS) was assessed on baseline NCCT and on follow-up dual-energy CT (SOMATOM Force, Siemens Healthcare GmbH, Erlangen, Germany) obtained 24 h after EVT using the original PC-ASPECTS methodology (10). In patients with available CTP imaging, a CTP-derived PC-ASPECTS was additionally calculated using an analogous 10-point topographic scale, subtracting 1 point for each predefined region showing infarct core on CTP (commercially available software VEOcore, VEObrain GmbH, Freiburg, Germany) (11). CTP-derived infarct core volume (CBF < 30%) and ischemic volume (Tmax >6 s) were additionally recorded.

All imaging and angiographic assessments, including occlusion segment classification and PC-ASPECTS calculation, were independently performed by two board-certificated neuroradiologists who were blinded to clinical outcomes (CD and YL, each with 7 years of neurointervention experience). Discrepancies were resolved by consensus.

### Endovascular treatment

The decision for EVT was based primarily on the severity of the neurological deficits and the extent of infarct demarcation on baseline CT. Endovascular thrombectomy was performed using contemporary thrombectomy techniques, including aspiration, stent retrievers, or combined approaches at the discretion of the treating neurointerventionalist. The intravenous thrombolysis prior to EVT was recorded.

Reperfusion was graded using the modified Treatment in Cerebral Infarction (mTICI) scale. Successful reperfusion was defined as mTICI 2b to mTICI 3. First pass effect was defined as complete reperfusion (mTICI 3) after the first pass.

### Clinical assessment and outcomes

Neurological status of patients at admission, during hospital stay, and at discharge was assessed using the NIHSS. To better capture clinically relevant deficit patterns beyond the total NIHSS score, NIHSS domain scores were additionally calculated to characterize segment-specific clinical presentation. This approach was chosen because the total NIHSS score reduces heterogenous neurological deficits to a single global value and may therefore reflect both the actual deficit pattern and early recovery only incompletely. For domain analyses, individual NIHSS items were grouped into predefined functional domains: consciousness (items 1a–1c), visual (items 2–3), facial palsy (item 4), motor (items 5a–6b), limb ataxia (item 7), sensory (item 8), language (items 9–10), and neglect (item 11). A domain was considered affected if any item within that domain was scored >0, and results are reported as proportions of patients with domain involvement at baseline and discharge.

Safety outcomes included intracranial hemorrhage assessed on 24 h dual-energy CT and in-hospital mortality.

### Statistical analysis

Continuous variables are presented as median with interquartile range (IQR) and were compared using the Mann–Whitney U test. Categorical variables are reported as counts and percentages and were compared using the *χ*^2^ or Fisher’s exact tests. All analyses were exploratory due to the limited sample size. Statistical significance was defined as a two-sided *p*-value <0.05. Statistical analyses were performed using SPSS Statistics (version 31, IBM Corp., Armonk, NY, USA).

## Results

### Study population and segment-specific baseline characteristics

Between January 2018 and October 2025, a total of 1,166 patients underwent EVT for acute ischemic stroke at a tertiary care center. Among these, 39 patients (3.3%) meeting inclusion criteria with an isolated P1 or P2 segment occlusion were included for final analysis. Occlusion site involved the P1 segment in 27 patients (69%) and P2 in 12 patients (31%).

Baseline characteristics stratified by occlusion segment are summarized in Table 1. Median age was comparable between patients with P1 and P2 occlusions (77 vs. 76 years, *p* = 1.0), as was the proportion of male patients (56% vs. 67%, *p* = 0.7). Pre-stroke disability did not differ significantly [modified Rankin Scale (mRS) 0 (0–2) vs. 1 (0–1), *p* = 0.8].

**Table 1:** Baseline characteristics stratified by occlusion segment.

Variable	Overall (*n* = 39)	P1 (*n* = 27)	P2 (*n* = 12)	*p* value
Age (years)	77 (63–83)	77 (59–83)	76 (63–85)	1.0
Male sex	23 (59%)	15 (55.6%)	8 (66.7%)	0.73
Pre-stroke mRS	0 (0–2)	0 (0–2)	1 (0–1)	0.82
Witnessed stroke	25 (64.1%)	17 (63%)	8 (66.7%)	1.0
Baseline NIHSS (points)	9 (6–21)	12 (7–23)	7 (4–9)	0.06
Baseline CT PC-ASPECTS (points)	10 (9–10)	9 (9–10)	10 (10–10)	0.06
Baseline CTP PC-ASPECTS (points)	10 (9–10), 6 missing	10 (8–10)	10 (9–10)	0.28
Baseline CTP Infarct Core Volume (ml)	0 (0–5)	0 (0–8)	0 (0–1)	0.27
Baseline CTP Ischemic Volume (ml)	34 (22–50)	35 (13–53)	34 (24–50)	0.81
Interval from symptom onset to admission (min)	119 (63–524)	139 (85–551)	75 (26–128)	0.056
Intravenous thrombolysis	24 (61.5%)	18 (66.7%)	6 (50%)	0.48
Diabetes mellitus	15 (38.5%)	13 (48.1%)	2 (16.7%)	0.08
Arterial hypertension	30 (76.9%)	21 (77.8%)	9 (75%)	1.0
Atrial fibrillation	21 (53.8%)	11 (40.7%)	10 (83.3%)	0.014*
Prior stroke	11 (28.2%)	9 (33.3%)	2 (16.7%)	0.45

P1/P2, segments of the posterior cerebral artery; mRS, modified Rankin Scale; NIHSS, National Institutes of Health Stroke Scale; PC, posterior circulation; ASPECTS, Alberta Stroke Program Early CT Score. *p*-values <0.05 are bolded and marked with the symbol *.

Median baseline NIHSS was markedly higher in patients with P1 occlusion [12 (7–23) vs. 7 (4–9)], showing a trend toward significance (*p* = 0.06). Baseline PC-ASPECTS [9 (9–10) vs. 10 (10–10)] as well as CTP PC-ASPECTS [10 (8–10) vs. 10 (9–10)] were similar in both groups. CTP-derived infarct core volume and ischemic volume did not differ significantly between groups. Time from symptom onset to admission was interestingly longer in P1 compared with P2 occlusions [139 (85–551) vs. 75 (26–128) minutes], showing a trend toward significance (*p* = 0.056).

Atrial fibrillation was significantly more frequent in P2 than in those with P1 occlusions (83% vs. 41%, *p* = 0.014). Other vascular risk factors, including hypertension, diabetes mellitus, and prior stroke, were similarly distributed between groups. Rates of intravenous thrombolysis were not significantly different (67% vs. 50%, *p* = 0.5).

### Domain-specific clinical presentation (baseline NIHSS)

Domain-specific NIHSS analysis revealed distinct clinical presentation patterns owing to occlusion site (Table 2). Patients with P1 occlusions more frequently exhibited motor deficits (100% vs. 58%, *p* = 0.004), sensory deficits (85% vs. 25%, *p* = 0.001), and facial palsy (74% vs. 25%, *p* = 0.012) compared with patients with P2 occlusions. In contrast, visual field deficits were common in both groups (82% vs. 67%). Language impairment and neglect were infrequent overall and showed no significant segment-specific differences (Table 2).

**Table 2:** NIHSS domain deficits at baseline (domain score > 0).

NIHSS domain	All (*n* = 39)	P1 (*n* = 27)	P2 (*n* = 12)	*p* value
Level of consciousness (items 1a-1c)	19 (48.7%)	13 (48.1%)	6 (50%)	0.92
Visual (items 2–3)	30 (76.9%)	22 (81.5%)	8 (66.7%)	0.67
Facial palsy (item 4)	23 (59.0%)	20 (74.1%)	3 (25%)	0.012*
Motor (items 5a-6b)	34 (87.2%)	27 (100%)	7 (58.3%)	0.004*
Limb ataxia (item 7)	11 (28.2%)	6 (22.2%)	5 (41.7%)	0.24
Sensory (item 8)	26 (66.7%)	23 (85.2%)	3 (25%)	0.001*
Language (items 9–10)	29 (74.4%)	22 (81.1%)	7 (58.3%)	0.4
Neglect (item 11)	9 (23.1%)	8 (29.6%)	1 (8.3%)	0.24

NIHSS domains were dichotomized as present (item score >0) or absent (item score = 0). NIHSS, National Institutes of Health Stroke Scale; P1/P2, segments of the posterior cerebral artery. *p*-values <0.05 are bolded and marked with the symbol *.

### Angiographic results

Procedural details are provided in Table 3. Median door-to-groin times [84 (74–117) vs. 91 (64–133) minutes] and groin-to-reperfusion times did not differ significantly between groups. Successful reperfusion (mTICI 2b–3) was achieved in 28 patients (72%) overall, occurring numerically more often in P1 than P2 occlusions (78% vs. 58%), although this difference was not statistically significant. First pass effect was observed in a minority of cases (23%) and did not differ between groups. EVT techniques used were comparable across segments (combined maneuver with stent-retriever and aspiration catheter in 70% of P1 occlusions, and 58% of P2 occlusions).

**Table 3:** Procedural characteristics and angiographic results.

Variable	Overall (*n* = 39)	P1 (*n* = 27)	P2 (*n* = 12)	*p* value
Door-to-groin puncture time (min)	84 (68–131)	84 (74–117)	91 (64–133)	0.82
Groin puncture-to-reperfusion time (min)	52 (37–68)	53 (44–72)	46 (31–62)	0.18
Aspiration	8 (20.5%)	5 (18.5%)	3 (25%)	0.664
Combination of Aspiration and Stent-Retriever	26 (66.7%)	19 (70.4%)	7 (58.3%)	
First pass effect	9 (23.1%)	8 (33.3%)	1 (9.1%)	0.22
mTICI 0–2a	11 (28.2%)	6 (22.2%)	5 (41.7%)	0.4
mTICI 2b	9 (23.1%)	6 (22.2%)	3 (25%)	
mTICI 2c-3	19 (48.8%)	15 (55.6%)	4 (33.3%)	
Successful reperfusion (mTICI 2b-3)	28 (71.8%)	21 (77.8%)	7 (58.3%)	0.26

P1/P2, segments of the posterior cerebral artery; mTICI, modified Treatment in Cerebral Infarction score.

### Early imaging and clinical outcomes

#### Follow-up imaging

On 24 h CT, PC-ASPECTS was similar in patients with P1 and P2 occlusions [both 8 (8–9), *p* = 0.9]. Regional PC-ASPECTS involvement at baseline and 24 h is summarized in Supplementary Table S1. At baseline CT, early ischemic change in thalamic region was observed in 15% of P1 occlusions and in none of the P2 occlusions, while occipital involvement was present in 37% vs. 17%, respectively. At 24 h, occipital involvement was frequent in both groups (59% vs. 64%), and thalamic involvement was observed in 41% (P1) vs. 36% (P2).

#### Neurological status and domain-specific residual deficits at discharge

The median reduction in NIHSS from admission to 24 h was comparable between groups (P1: −1 [IQR −6 to 2] vs. P2: −2 [IQR −6 to 2], *p* = 0.7). Domain-specific deficits at discharge are detailed in Table 4. Compared with P2 occlusions, P1 occlusions more frequently exhibited persistent deficits in consciousness (45% vs. 0%, *p* = 0.027), motor function (80% vs. 33%, *p* = 0.032), sensory deficits (55% vs. 0%, *p* = 0.005), and language impairment (75% vs. 33%, *p* = 0.048). Visual deficits remained common without significant segment-specific differences (70% vs. 44%, *p* = 0.2).

**Table 4:** Radiological and clinical outcomes.

Outcome	Overall (*n* = 39)	P1 (*n* = 27)	P2 (*n* = 12)	*p* value
Any intracranial hemorrhage	5 (12.8%)	4 (14.8%)	1 (9.1%)	1.0
24-h PC-ASPECTS	8 (8–9)	8 (8–9)	8 (8–9)	0.92
NIHSS at discharge	8 (3–28)	12 (6–28)	3 (1–32)	0.08
NIHSS change (admission to discharge)	−1 [(−6) to 2]	−1 [(−6) to 2]	−2 [(−6) to 2]	0.71
Hospital stay (days)	9 (5–13)	9 (3–13)	9 (6–17)	0.48
In-hospital mortality	10 (25.6%)	7 (25.9%)	3 (25%)	1.0
Level of consciousness (items 1a-1c)	9 (31%)	9 (45%)	0	0.027*
Visual (items 2–3)	18 (62.1%)	14 (70%)	4 (44.4%)	0.24
Facial palsy (item 4)	16 (55.2%)	13 (65%)	3 (33.3%)	0.23
Motor (items 5a-6b)	19 (65.5%)	16 (80%)	3 (33.3%)	0.032*
Limb ataxia (item 7)	5 (17.2%)	3 (15%)	2 (22.2%)	0.63
Sensory (item 8)	11 (37.9%)	11 (55%)	0	0.005*
Language (items 9–10)	18 (62.1%)	15 (75%)	3 (33.3%)	0.048*
Neglect (item 11)	10 (34.5%)	8 (40%)	2 (22.2%)	0.431

Negative values of NIHSS change indicate a reduction.

PC, posterior circulation; ASPECTS, Alberta Stroke Program Early CT Score; NIHSS, National Institutes of Health Stroke Scale; P1/P2, segments of the posterior cerebral artery. *p*-values <0.05 are bolded and marked with the symbol *.

Any intracranial hemorrhage on follow-up imaging occurred in 13% and did not differ significantly between P1 and P2 occlusions. In-hospital mortality was 26% overall and similar in both groups. In-hospital mortality was largely driven by severe neurological primary injury or malignant posterior infarction (6/10) as well as severe comorbidities (3/10) rather than the procedure itself, with only one fatal case being primarily procedure-related. Length of hospital stay was similar in both groups (median 9 days).

#### Exploratory analysis according to reperfusion status and occlusion location

In P1 occlusions, 24 h PC-ASPECTS did not differ significantly between successful and unsuccessful reperfusion [8 (8–9) vs. 8 (8–8)], and NIHSS reduction from admission to discharge was similar [−1 (−7 to 2) vs. −1 (−3 to 5); Supplementary Table S2]. However, regional follow-up imaging suggested more frequent infarct demarcation in unsuccessful P1 reperfusion, particularly involving the thalamus (83% vs. 29%) and occipital lobe (83% vs. 52%, Supplementary Table S1).

**Figure 3:**
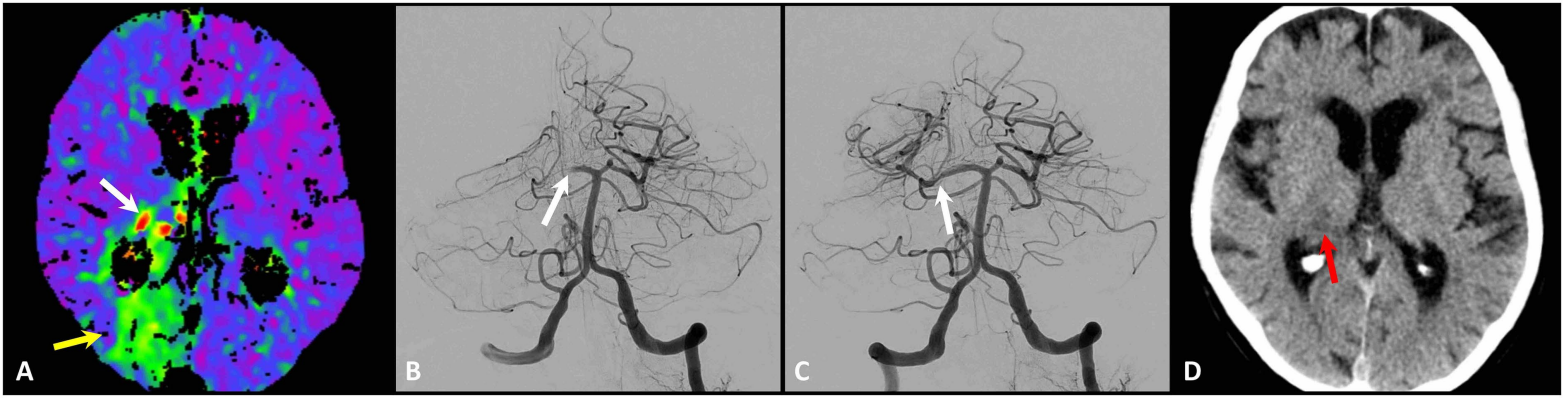
Illustrative case example of a patient with ischemic stroke due to P2 occlusion. A 91-year-old female presented with visual field deficit (partial left hemianopia), mild left-sided facial weakness and hemiparesis, limb ataxia, sensory deficit, and dysarthria. Baseline CT perfusion demonstrates hypoperfusion in the right occipital lobe (yellow arrow) involving also the right thalamic margin possibly caused by occlusion of thalamogeniculate artery (white arrow) **(A)**. DSA confirms a P2 occlusion (arrow) in the front view **(B)**. Post-thrombectomy control shows complete reperfusion (mTICI 3) of the PCA territory with mild vasospasm (arrow) of P2 segment after clot retrieval **(C)**. Concordant to CT perfusion, 24 h postinterventional CT demonstrates a focal thalamic infarction (red arrow) despite complete angiographic reperfusion after a time window of 1,305 min since last seen well **(D)**. PCA, posterior cerebral artery; DSA, digital subtraction angiography; P2, segment of the PCA; mTICI, modified Treatment in Cerebral Infarction.

In P2 occlusions, differences by reperfusion status were less pronounced. PC-ASPECTS at 24 h was numerically higher after successful reperfusion [9 (8–9) vs. 8 (7–9)], while NIHSS reduction from admission to discharge did not differ significantly [−2 (−4 to 2) vs. −6 (−8 to 19); Supplementary Table S2]. Regional follow-up imaging showed only modest differences between successful and unsuccessful reperfusion for occipital (57% vs. 60%) and thalamic involvement (29% vs. 40%, Supplementary Table S1; Figure 3). Domain-specific discharge deficits by reperfusion status are shown in Supplementary Table S3.

## Discussion

In this retrospective single-center exploratory cohort of EVT-treated isolated PCA occlusions, we observed segment-specific differences in baseline presentation and comorbidity patterns between P1 and P2 occlusions, while EVT was technically feasible across segments. Patients with P1 occlusion presented with more severe neurological impairment than those with P2 occlusion (NIHSS 12 vs. 7), with significantly more frequent motor and sensory deficits and facial palsy. In contrast, P2 occlusions more often presented with comparatively lower NIHSS despite a high prevalence of visual dysfunction. Although successful reperfusion was more frequent in P1 occlusions, substantial neurological impairment frequently persisted at discharge, particularly in motor, sensory, vigilance, and language-related domains. This underscores that successful reperfusion does not necessarily translate into clinical recovery in P1 occlusions. At the tissue level, however, successful reperfusion in P1 was associated with a lower frequency of infarct demarcation in deep and cortical PCA territories, most notably thalamic and occipital involvement. These findings add segment-level granularity to classic PCA territory cohorts demonstrating heterogeneity in phenotype, mechanism, and outcome (12–15).

The segment-dependent phenotype observed in our cohort is anatomically plausible and can be interpreted in the context of the anatomical supply patterns illustrated in Figure 2. Microanatomical descriptions emphasize that the PCA’s proximal and distal segments have distinct relationships to deep and cortical territories (9). Proximal occlusion may be more likely to affect thalamopeduncular networks either through perforator vulnerability or collateral constraints, thereby producing broader neurological deficits including vigilance reduction and motor/sensory involvement, as reflected in our P1 domain profile. Conversely, P2 occlusions preferentially map to cortical PCA territory and inferomedial temporal regions, coherently explaining the predominance of visual syndromes and the comparatively lower NIHSS. This anatomical–clinical heterogeneity is consistently emphasized in posterior circulation stroke reviews (16).

Our results further illustrate that angiographic macro-reperfusion does not necessarily prevent deep territory injury in posterior circulation stroke. Despite a high overall rate of successful reperfusion (72%), deep-territory infarction remained frequent on 24-h CT imaging, including thalamic infarction in 41% (P1) and 36% (P2). In P1 occlusions, exploratory analyses suggested that unsuccessful reperfusion was associated with higher rates of thalamic and occipital lobe infarct demarcation at 24 h, and discharge NIHSS domains similarly indicated a less favorable residual deficit profile, particularly for impaired consciousness, language deficits, and neglect. In P2 occlusions, differences by reperfusion status appeared less pronounced. However, P2 occlusions also showed a lower reperfusion rate, which may reflect the greater technical complexity of distal PCA thrombectomy, including smaller vessel caliber, more difficult distal access, and branch angulation. Potential explanations for deep territorial injury despite successful reperfusion include early irreversible tissue injury before reperfusion, impaired collateral supply, distal embolization into small branches, or microvascular no-reflow (17–19). These mechanisms may be particularly relevant in P1 occlusions, where reperfusion of perforator-dependent deep territories may remain incomplete despite successful parent-vessel recanalization, whereas P2 occlusions more often affect cortical territories (Figure 2). Recent meta-analyses echo this mixed picture, reporting early neurological gains and high recanalization feasibility but inconsistent functional superiority and variable safety signals (20–23). Within this landscape, our segment-focused observations suggest that outcomes and clinical presentation may vary by occlusion segment, and that segment-level phenotyping should be considered in future observational studies and prospective trials.

An additional implication of our domain-level analysis is that P1 and P2 occlusions may differ in how “visible” their deficits are to NIHSS. This aligns with prior work indicating that baseline NIHSS behaves differently in anterior versus posterior circulation strokes (24) and with the rationale for posterior-adapted severity measures (6). Clinically, this matters because distal PCA occlusions may produce disabling visual–cognitive deficits that are incompletely reflected by global NIHSS, whereas proximal occlusions more often include deficits that drive NIHSS upward. Such phenotypic variability was described in prior studies of superficial PCA territory infarction (25, 26) and supports the value of segment- and domain-level characterization when interpreting severity and treatment eligibility in isolated PCA occlusion. In parallel, we observed longer symptom onset–to-admission time in P1 occlusions than in P2 occlusions. This finding is consistent with the literature showing that posterior circulation strokes are disproportionately missed or diagnosed late, especially when symptoms are atypical compared to anterior circulation ischemic stroke (4, 5, 27). Proximal PCA involvement may affect neuronal networks relevant to arousal/attention, medial temporal memory circuits, and precuneus-related visuospatial/cognitive function (28), potentially complicating symptom recognition and onset ascertainment and thereby contributing to prolonged time from symptom-onset to hospital admission.

The markedly higher prevalence of atrial fibrillation among P2 occlusions (83% vs. 41%) supports the higher rates of possible embolic contribution to distal PCA occlusions, whereas P1 occlusions may represent a broader etiologic spectrum, including underlying arteriosclerotic vessel change as reflected by higher prevalence of diabetes mellitus. This interpretation aligns with a previous study describing heterogeneity of infarction mechanisms in superficial PCA territory infarction (29) and is consistent with studies emphasizing that PCA vascular variants can modulate both tissue-at-risk and collateral supply (30–32). Segment-level phenotyping may therefore be relevant not only for clinical presentation but also for etiologic stratification and treatment decision-making in isolated PCA occlusion.

This study has several limitations. The retrospective single-center design introduces the risk of selection bias and unmeasured confounding. The limited sample size, particularly in the P2 subgroup, substantially restricts statistical power and limits generalizability. The present findings should therefore be considered exploratory rather than confirmatory. Follow-up was restricted to NIHSS as an in-hospital neurological outcome surrogate. Long-term functional outcome (90-day mRS) and structured visual and neurocognitive assessments were not systematically available, which is particularly relevant in PCA stroke, where disability may be driven by domains not fully captured by NIHSS-based surrogates. Finally, treatment strategy and device choice were not standardized and reflected operator discretion, limiting inference regarding technique-specific effects.

## Conclusion

In EVT-treated isolated PCA occlusion, P1 and P2 segments show distinct clinical presentation profiles and potentially different etiologic patterns. P1 occlusion is frequently associated with motor and sensory deficits in addition to visual dysfunction, whereas P2 occlusion more often presents with predominantly visual impairment and lower NIHSS. EVT was technically feasible across segments with acceptable safety in this real-world cohort. However, substantial neurological impairment often persisted at discharge despite successful reperfusion, highlighting the complex relationship between angiographic success, tissue injury and neurological recovery in PCA stroke. Future multicenter studies should incorporate segment-specific phenotyping to refine patient selection and clarify the clinical benefit of EVT.

## Data Availability

The raw data supporting the conclusions of this article will be made available by the authors, without undue reservation.

## References

[1] Ntaios G Spengos K Vemmou A Savvari P Koroboki E Stranjalis G . Long-term outcome in posterior cerebral artery stroke. Eur J Neurol. (2011) 18:1074–80. doi: 10.1111/j.1468-1331.2011.03384.x, 21435108

[2] Duloquin G Graber M Garnier L Crespy V Comby P-O Baptiste L . Incidence of acute ischemic stroke with visible arterial occlusion. Stroke. (2020) 51:2122–30. doi: 10.1161/STROKEAHA.120.029949 32486967

[3] Maulaz AB Bezerra DC Bogousslavsky J. Posterior cerebral artery infarction from middle cerebral artery infarction. Arch Neurol. (2005) 62:938–41. doi: 10.1001/archneur.62.6.938, 15956164

[4] Arch AE Weisman DC Coca S Nystrom KV Wira CR Schindler JL. Missed ischemic stroke diagnosis in the emergency department by emergency medicine and neurology services. Stroke. (2016) 47:668–73. doi: 10.1161/STROKEAHA.115.010613, 26846858

[5] Gurley KL Edlow JA. Avoiding misdiagnosis in patients with posterior circulation ischemia: a narrative review. Acad Emerg Med. (2019) 26:1273–84. doi: 10.1111/acem.13830, 31295763

[6] Alemseged F Rocco A Arba F Schwabova JP Wu T Cavicchia L . Posterior National Institutes of Health stroke scale improves prognostic accuracy in posterior circulation stroke. Stroke. (2022) 53:1247–55. doi: 10.1161/STROKEAHA.120.034019, 34905944

[7] Nguyen TN Qureshi MM Strambo D Strbian D Räty S Herweh C . Endovascular versus medical management of posterior cerebral artery occlusion stroke: the PLATO study. Stroke. (2023) 54:1708–17. doi: 10.1161/STROKEAHA.123.042674, 37222709

[8] Meyer L Stracke CP Jungi N Wallocha M Broocks G Sporns PB . Thrombectomy for primary distal posterior cerebral artery occlusion stroke: the TOPMOST study. JAMA Neurol. (2021) 78:434–44. doi: 10.1001/jamaneurol.2021.0001 33616642 PMC7900924

[9] Zeal AA Rhoton AL. Microsurgical anatomy of the posterior cerebral artery. J Neurosurg. (1978) 48:534–59. doi: 10.3171/jns.1978.48.4.0534, 632878

[10] Puetz V Sylaja PN Coutts SB Hill MD Dzialowski I Mueller P . Extent of hypoattenuation on CT angiography source images predicts functional outcome in patients with basilar artery occlusion. Stroke. (2008) 39:2485–90. doi: 10.1161/strokeaha.107.511162, 18617663

[11] Psychogios M-N Sporns PB Ospel J Katsanos AH Kabiri R Flottmann FA . Automated perfusion calculations vs. visual scoring of collaterals and CBV-ASPECTS. Clin Neuroradiol. (2021) 31:499–506. doi: 10.1007/s00062-020-00974-3 33216157 PMC8211603

[12] Fisher C. The posterior cerebral artery syndrome. Can J Neurol Sci. (1986) 13:232–9. doi: 10.1017/s0317167100036337, 3742339

[13] Yamamoto Y Georgiadis AL Chang H-M Caplan LR. Posterior cerebral artery territory infarcts in the New England medical center posterior circulation registry. Arch Neurol. (1999) 56:824–32. doi: 10.1001/archneur.56.7.824, 10404984

[14] Brandt T Steinke W Thie A Pessin MS Caplan LR. Posterior cerebral artery territory infarcts: clinical features, infarct topography, causes and outcome: multicenter results and a review of the literature. Cerebrovasc Dis. (2000) 10:170–82. doi: 10.1159/000016053 10773642

[15] Arboix A Arbe G García-Eroles L Oliveres M Parra O Massons J. Infarctions in the vascular territory of the posterior cerebral artery: clinical features in 232 patients. BMC Res Notes. (2011) 4:329. doi: 10.1186/1756-0500-4-329, 21899750 PMC3180463

[16] Salerno A Strambo D Nannoni S Dunet V Michel P. Patterns of ischemic posterior circulation strokes: a clinical, anatomical, and radiological review. Int J Stroke. (2022) 17:714–22. doi: 10.1177/17474930211046758, 34581223 PMC9358301

[17] Sperring CP Savage WM Argenziano MG Leifer VP Alexander J Echlov N . No-reflow post-recanalization in acute ischemic stroke: mechanisms, measurements, and molecular markers. Stroke. (2023) 54:2472–80. doi: 10.1161/STROKEAHA.123.044240, 37534511

[18] Liebeskind DS Jahan R Nogueira RG Jovin TG Lutsep HL Saver JL. Early arrival at the emergency department is associated with better collaterals, smaller established infarcts and better clinical outcomes with endovascular stroke therapy: SWIFT study. J Neurointerv Surg. (2016) 8:553–8. doi: 10.1136/neurintsurg-2015-011758, 25964375

[19] Yeo LLL Holmberg A Mpotsaris A Söderman M Holmin S Kuntze Söderqvist A . Posterior circulation occlusions may be associated with distal emboli during thrombectomy: factors for distal embolization and a review of the literature. Clin Neuroradiol. (2019) 29:425–33. doi: 10.1007/s00062-018-0679-z, 29569010 PMC6710331

[20] Alkhiri A Alamri AF Alharbi AR Almaghrabi AA Alansari N Niaz AA . Endovascular therapy versus best medical management for isolated posterior cerebral artery occlusion: a systematic review and meta-analysis. Eur Stroke J. (2024) 9:69–77. doi: 10.1177/23969873231201715, 37752802 PMC10916830

[21] Jhou HJ Lee CH Tsai YC Chen PH Yang LY. Is thrombectomy worth it for isolated posterior cerebral artery occlusion? Meta-analysis and trial sequential analysis. Stroke Vasc Interv Neurol. (2024) 4:e001084. doi: 10.1161/SVIN.123.001084 41583603 PMC12778484

[22] Moawad MHED Gadelmawla AF Abu Alia LA Abdul-Hafez HA Alkhawaldeh IM Mohammed MK . Endovascular therapy versus medical management in posterior cerebral artery stroke: neurological gains without functional superiority: a meta-analysis. BMC Neurol. (2025) 25:508. doi: 10.1186/s12883-025-04512-x, 41402772 PMC12709783

[23] Alam U Ahmed R Rath S Khattak F Asif MA Alatise MB . Endovascular therapy versus best medical treatment in posterior cerebral artery stroke: a systematic review and meta-analysis. Brain Behav. (2026) 16:e71194. doi: 10.1002/brb3.71194, 41499298 PMC12778430

[24] Sato S Toyoda K Uehara T Toratani N Yokota C Moriwaki H . Baseline NIH stroke scale score predicting outcome in anterior and posterior circulation strokes. Neurology. (2008) 70:2371–7. doi: 10.1212/01.wnl.0000304346.14354.0b 18434640

[25] Cals N Devuyst G Afsar N Karapanayiotides T Bogousslavsky J. Pure superficial posterior cerebral artery territory infarction in the Lausanne stroke registry. J Neurol. (2002) 249:855–61. doi: 10.1007/s00415-002-0742-0, 12140669

[26] Benke T Dazinger F Pechlaner R Willeit K Clausen J Knoflach M. Lesion topography of posterior cerebral artery infarcts. J Neurol Sci. (2021) 428:117585. doi: 10.1016/j.jns.2021.117585, 34371243

[27] Hoyer C Szabo K. Pitfalls in the diagnosis of posterior circulation stroke in the emergency setting. Front Neurol. (2021) 12:682827. doi: 10.3389/fneur.2021.682827, 34335448 PMC8317999

[28] Kumral E Bayam FE Özdemir HN. Cognitive and behavioral disorders in patients with precuneal infarcts. Eur Neurol. (2021) 84:157–67. doi: 10.1159/000513098, 33827093

[29] Steinke W Mangold J Schwartz A Hennerici M. Mechanisms of infarction in the superficial posterior cerebral artery territory. J Neurol. (1997) 244:571–8. doi: 10.1007/s004150050146, 9352455

[30] Ryu J-C Kim JS. Mechanisms of stroke in patients with fetal posterior cerebral artery. J Stroke Cerebrovasc Dis. (2022) 31:106518. doi: 10.1016/j.jstrokecerebrovasdis.2022.106518, 35605387

[31] Dharmasaroja PA Uransilp N Piyabhan P. Fetal origin of posterior cerebral artery related to poor collaterals in patients with acute ischemic stroke. J Clin Neurosci. (2019) 68:158–61. doi: 10.1016/j.jocn.2019.07.006, 31337580

[32] Schomer DF Marks MP Steinberg GK Johnstone IM Boothroyd DB Ross MR . The anatomy of the posterior communicating artery as a risk factor for ischemic cerebral infarction. N Engl J Med. (1994) 330:1565–70. doi: 10.1056/NEJM199406023302204, 8177246

[33] Li S Kumar Y Gupta N Abdelbaki A Sahwney H Kumar A . Clinical and neuroimaging findings in thalamic territory infarctions: a review. J Neuroimaging. (2018) 28:343–9. doi: 10.1111/jon.12503, 29460331

