# Supplementary material for: Endovascular thrombectomy for isolated posterior cerebral artery occlusion: distinct clinical presentation patterns and neurological outcomes in P1 versus P2 segments
Source: Front Neurol. 2026 Apr 21;17:1807618. doi: 10.3389/fneur.2026.1807618 (PMC13141681; doi:10.3389/fneur.2026.1807618)
Supplement: Supplementary file 1 [file Table_1.docx]

## Supplementary Material:

Manuscript Title: Endovascular Thrombectomy for Isolated Posterior Cerebral Artery Occlusion: Distinct Clinical Presentation Patterns and Neurological Outcomes in P1 versus P2 Segments

**Table S1:** Regional PC-ASPECTS involvement at baseline and at 24 hours by reperfusion status (TICI 2b–3 vs. ≤2a), stratified by occlusion segment (P1 vs. P2)

| **Baseline PC ASPECTS** | **P1 (n=21), successful** | **P1 (n=6), unsuccessful** | **P2 (n=7), successful** | **P2 (n=5), unsuccessful** |
| --- | --- | --- | --- | --- |
| Cerebellum (left or right) | 2 (9.5%) | 0 | 0 | 0 |
| Middle Brain | 1 (4.8%) | 0 | 0 | 0 |
| Pons | 0 | 0 | 0 | 0 |
| Occipital Lobe (left or right) | 8 (38.1%) | 2 (33.3%) | 2 (28.6%) | 0 |
| Thalamus | 2 (9.5%) | 2 (33.3%) | 0 | 0 |
| **24h PC ASPECTS** | **P1 (n=21), successful** | **P1 (n=6), unsuccessful** | **P2 (n=7), successful** | **P2 (n=5), unsuccessful** |
| Cerebellum (left or right) | 5 (23.8%) | 0 | 3 (42.9%) | 1 (20%) |
| Middle Brain | 6 (28.6%) | 0 | 0 | 1 (20%) |
| Pons | 3 (14.3%) | 0 | 0 | 0 |
| Occipital Lobe (left or right) | 11 (52.4%) | 5 (83.3%) | 4 (57.1%) | 3 (60%) |
| Thalamus | 6 (28.6%) | 5 (83.3%) | 2 (28.6%) | 2 (40%) |

**Table S2:** Global clinical and imaging outcomes by reperfusion status (TICI 2b–3 vs. ≤2a), stratified by occlusion segment (P1 vs. P2)

|  | **P1 (n=27)** | | **P2 (n=12)** | |
| --- | --- | --- | --- | --- |
|  | **Successful reperfusion (n=21), 6 died** | **Unsuccessful reperfusion (n=6), 1 died** | **Successful reperfusion (n=7), 2 died** | **Unsuccessful reperfusion (n=5), 1 died** |
| Baseline NIHSS (points) | 12 (7-24) | 11 (8-25) | 8 (4-38) | 6 (5-9) |
| Discharge NIHSS | 12 (6-31) | 16 (6-29) | 3 (2-40) | 2 (0-24) |
| NIHSS Change (admission to discharge) | -1 (-7 to 2) | -1 (-3 to 5) | -2 (-4 to 2) | -6 (-8 to 19) |
| Baseline PC-ASPECTS | 10 (9-10) | 10 (8-10) | 10 (9-10) | 10 (10-10) |
| 24h PC-ASPECTS | 8 (8-9) | 8 (8-8) | 9 (8-9) | 8 (7-9) |

**Table S3:** NIHSS domain involvement at baseline and discharge by reperfusion status (TICI 2b–3 vs. ≤2a), stratified by occlusion segment (P1 vs. P2)

| **Domains at Baseline** | **P1 successful reperfusion (n=21)** | **P1 unsuccessful reperfusion (n=6)** | **P2 successful reperfusion (n=7)** | **P2 unsuccessful reperfusion (n=5)** |
| --- | --- | --- | --- | --- |
| Level of consciousness (items 1a-1c) | 11 (52.4%) | 2 (33.3%) | 3 (42.9%) | 3 (60%) |
| Visual (items 2-3) | 16 (76.2%) | 6 (100%) | 6 (85.7%) | 2 (40%) |
| Facial palsy (item 4) | 15 (71.4%) | 5 (83.3%) | 3 (42.9%) | 0 |
| Motor (items 5a-6b) | 21 (100%) | 6 (100%) | 4 (57.1%) | 3 (60%) |
| Limb ataxia (item 7) | 6 (28.6%) | 6 (100%) | 2 (28.6%) | 3 (60%) |
| Sensory (item 8) | 18 (85.7%) | 5 (83.3%) | 3 (42.9%) | 0 |
| Language (items 9-10) | 17 (81%) | 5 (83.3%) | 3 (42.9%) | 4 (80%) |
| Neglect (item 11) | 7 (33.3%) | 1 (16.7%) | 1 (14.3%) | 0 |
| **Domains at Discharge** | **P1 successful reperfusion (n=15), 6 died** | **P1 unsuccessful reperfusion (n=5), 1 died** | **P2 successful reperfusion (n=5), 2 died** | **P2 unsuccessful reperfusion (n=4), 1 died** |
| Level of consciousness (items 1a-1c) | 6 (40%) | 3 (60%) | 0 | 0 |
| Visual (items 2-3) | 10 (66.7%) | 4 (80%) | 3 (60%) | 1 (25%) |
| Facial palsy (item 4) | 9 (60%) | 4 (80%) | 2 (40%) | 1 (25%) |
| Motor (items 5a-6b) | 12 (80%) | 4 (80%) | 2 (40%) | 1 (25%) |
| Limb ataxia (item 7) | 3 (20%) | 0 | 1 (20%) | 1 (25%) |
| Sensory (item 8) | 8 (53.3%) | 3 (60%) | 0 | 0 |
| Language (items 9-10) | 10 (66.7%) | 5 (100%) | 1 (20%) | 2 (50%) |
| Neglect (item 11) | 5 (33.3%) | 3 (60%) | 2 (40%) | 0 |
